# Supplementary material for: The industrial anaerobe Clostridium acetobutylicum uses polyketides to regulate cellular differentiation
Source: Nat Commun. 2017 Nov 15;8:1514. doi: 10.1038/s41467-017-01809-5 (PMC5686105; doi:10.1038/s41467-017-01809-5)
Supplement: Supplementary file 1 — Supplementary Information [file 41467_2017_1809_MOESM1_ESM.pdf]

a

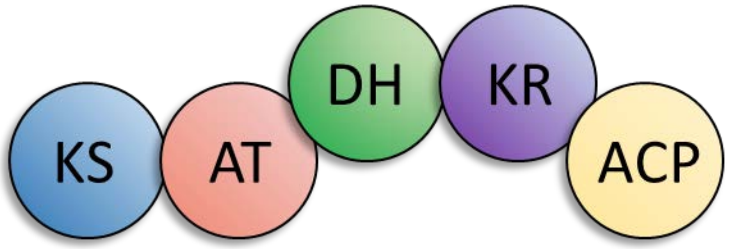

Conserved residue position	11	63	90	91	92	93	94	117	200	201	231	250	255
malonate consensus	Q	Q	G	H	S	LVIFAM	G	R	FP	H	ANTGEDS	NHQ	V
methylmalonate consensus	Q	Q	G	H	S	QMI	G	R	S	H	T	NS	V
PKS AT domain	I	Q	G	H	S	V	G	R	Y	H	T	N	V

b

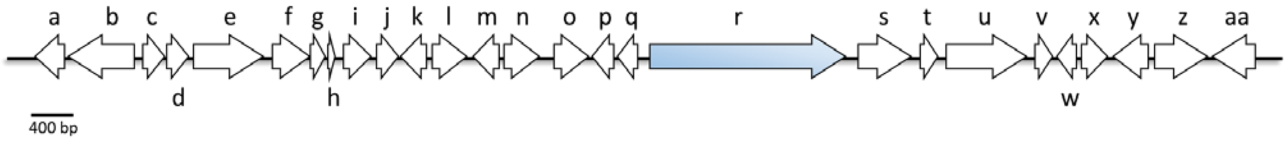

Label	Locus tag	Gene annotation	Label	Locus tag	Gene annotation
a	ca_c3372	conserved hypothetical protein	o	ca_c3358	site-specific DNA methylase
b	ca_c3371	oxidoreductase	p	ca_c3357	hypothetical protein
c	ca_c3370	transcriptional regulator	q	ca_c3356	lactoylglutathione lyase family protein
d	ca_c3369	transcriptional regulator	r	ca_c3355	type I polyketide synthase
e	ca_c3368	MFS transporter	s	ca_c3354	sodium ion-driven multidrug efflux pump
f	ca_c3367	HSP60 chaperonin family	t	ca_c3353	hypothetical protein
g	ca_c3366	hypothetical protein	u	ca_c3352	methyl-accepting chemotaxis protein
h	ca_c3365	hypothetical protein	v	ca_c3351	putative membrane protein
i	ca_c3364	ribonuclease inhibitor	w	ca_c3350	putative intracellular protease/amidase
j	ca_c3363	hypothetical protein	x	ca_c3349	LysR family transcriptional regulator
k	ca_c3362	conserved hypothetical protein	y	ca_c3348	homocysteine methyltransferase
l	ca_c3361	LysR family transcriptional regulator	z	ca_c3347	amino acid transporter
m	ca_c3360	LysR family transcriptional regulator	aa	ca_c3346	MFS transporter
n	ca_c3359	nitroreductase			

Supplementary Figure 1. Bioinformatic analysis of *pks* gene and gene neighborhood. (**a**) Upper: Representation of predicted PKS protein showing individual catalytic domains. KS, ketosynthase; AT, acyltransferase; DH, dehydratase; KR, ketoreductase; ACP, acyl carrier protein. Lower: Prediction of PKS AT domain substrate specificity based on previously established consensus sequences for malonate or methylmalonate substrates[1,2]. (**b**) Gene neighborhood surrounding *ca_c3355* on the *C. acetobutylicum* ATCC 824 genome (NCBI accession NC_003030.1). Gene annotations based on NCBI designations.

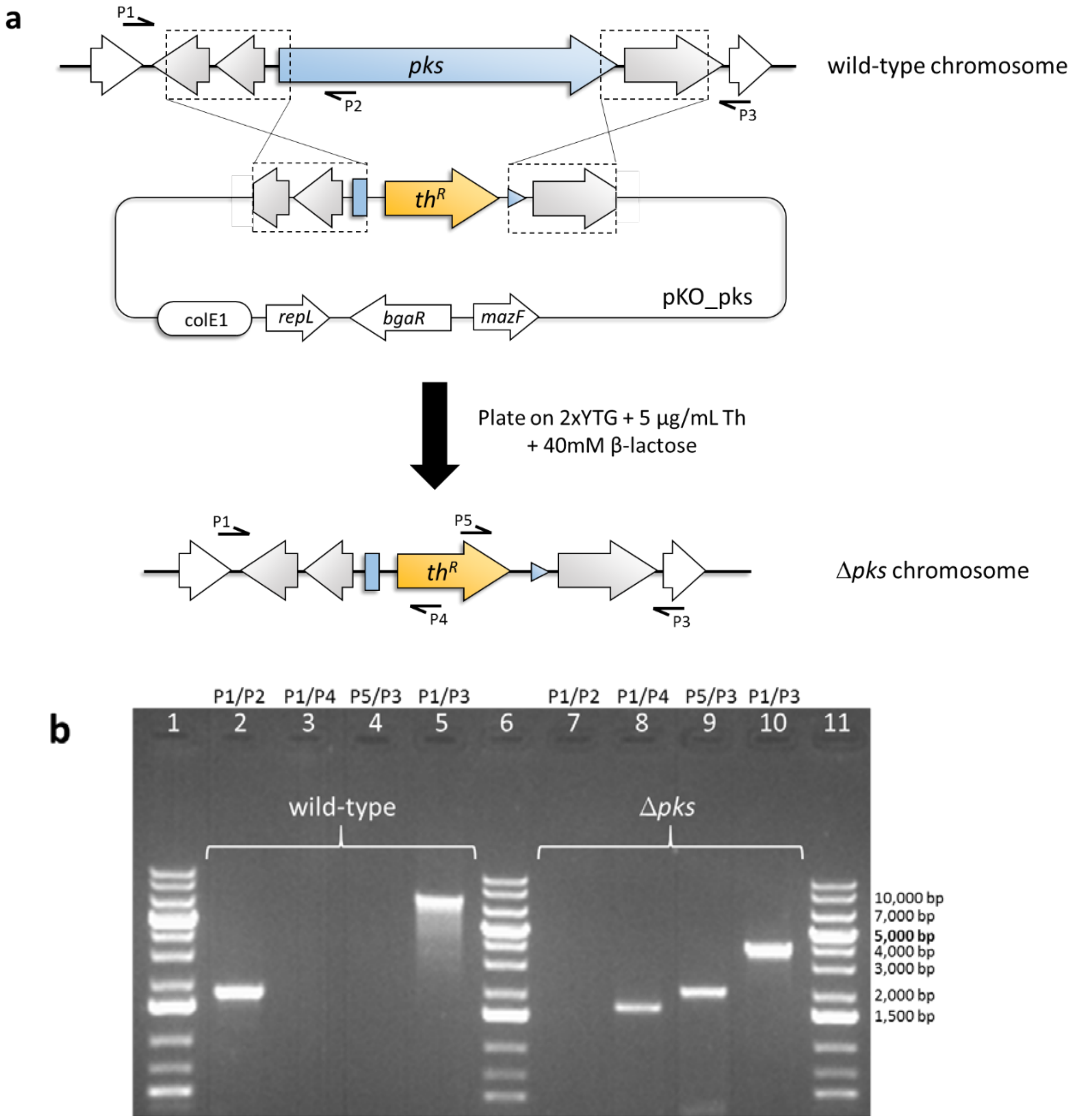

Supplementary Figure 2. Procedure for deletion of *pks* gene and verification of Δ*pks* by colony PCR. (**a**) Diagram of homologous recombination-based allelic exchange method used for deletion of the *C. acetobutylicum* pks gene. Dotted line boxes represent regions of homology surrounding the *pks* gene on the wild-type chromosome. Crosses between boxes represent homologous recombination events taking place between the wild-type chromosome and plasmid pKO_pks. Following plating on solid 2xYTG + 5 μg/mL Th + 40 mM β-lactose, only cells which have undergone the double crossover homologous recombination event (and lack plasmid pKO_pks) should be allowed to survive. th^R, thiamphenicol resistance gene; colE1, gram-negative origin of replication; *repL*, gram-positive replication protein; *bgaR*, repressor of promoter P_{bgaL}; *mazF*, toxin gene driven by promoter P_{bgaL}. Single barbed arrows represent primer annealing sites. (**b**) DNA electrophoresis gel of colony PCR reactions confirming deletion of *ca_c3355* (*pks*) in *C. acetobutylicum*. Lanes 2-5 show results for a wild-type colony template, while lanes 7-10 show results for a Δ*pks* colony template. Lanes 1, 6, and 11: GeneRuler™ 1 kb Plus ladder (Invitrogen, Thermo Fisher Scientific). Lanes 2 and 7: primers P1 & P2 (expected size for wild-type: 1.8 kb, expected size for Δ*pks*: no band). Lanes 3 and 8: primers P1 & P4 (expected size for wild-type: no band, expected size for Δ*pks*: 1.7 kb). Lanes 4 and 9: primers P5 & P3 (expected size for wild-type: no band, expected size for Δ*pks*: 2.1 kb). Lanes 5 and 10: primers P1 & P3 (expected size for wild-type: 8.2 kb, expected size for Δ*pks*: 4.0 kb).

a

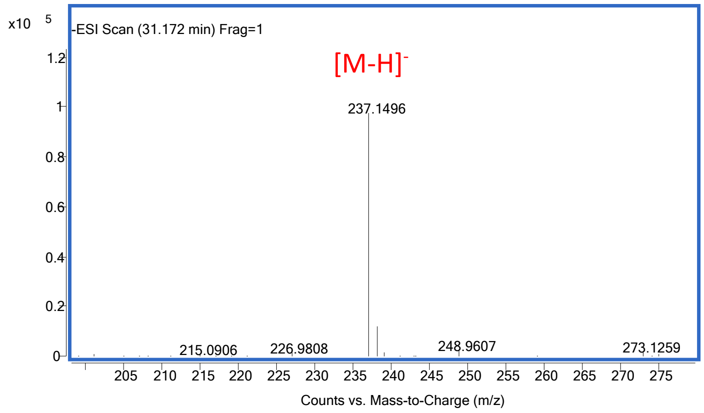

b

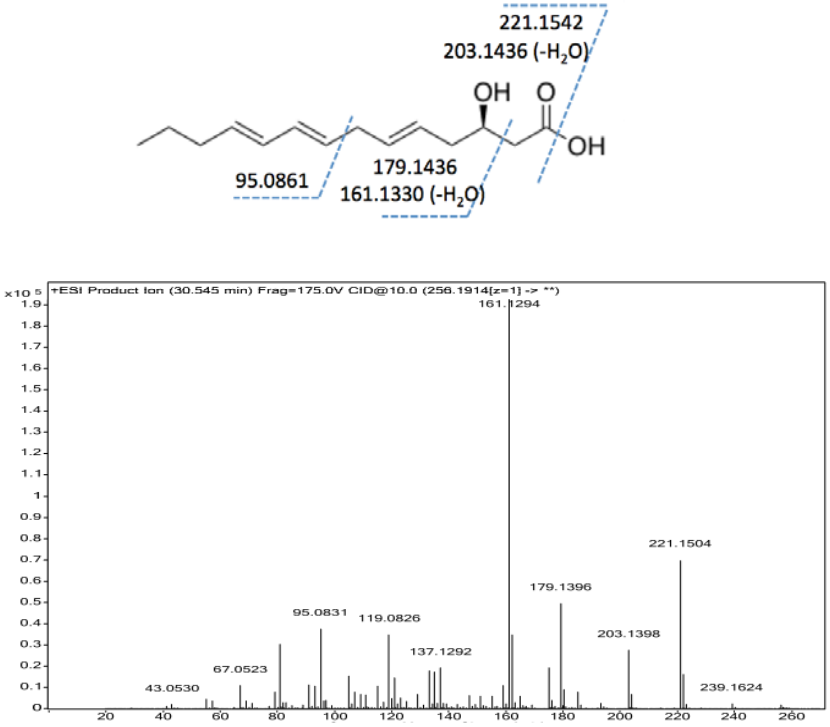

c

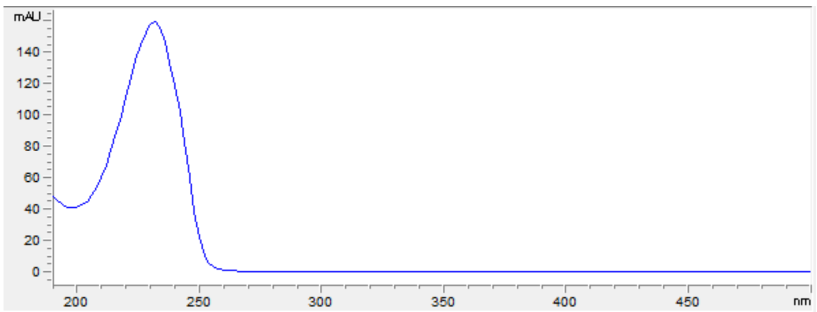

Supplementary Figure 3. HRMS and UV characterization of compound **1.** (**a**) (-)-HRMS (negative mode) of **1** (calc., 237.1496). (**b**) MS/MS analysis (positive mode) of **1**. (**c**) UV spectrum of **1**.

a

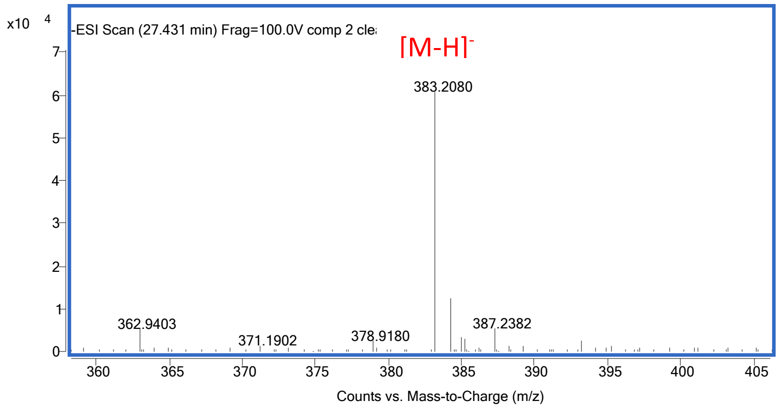
x10 4
-ESI Scan (27.431 min) Frag=100.0V comp 2 cle
[M-H]-
383.2080
362.9403
371.1902
378.9180
387.2382
Counts vs. Mass-to-Charge (m/z)

b

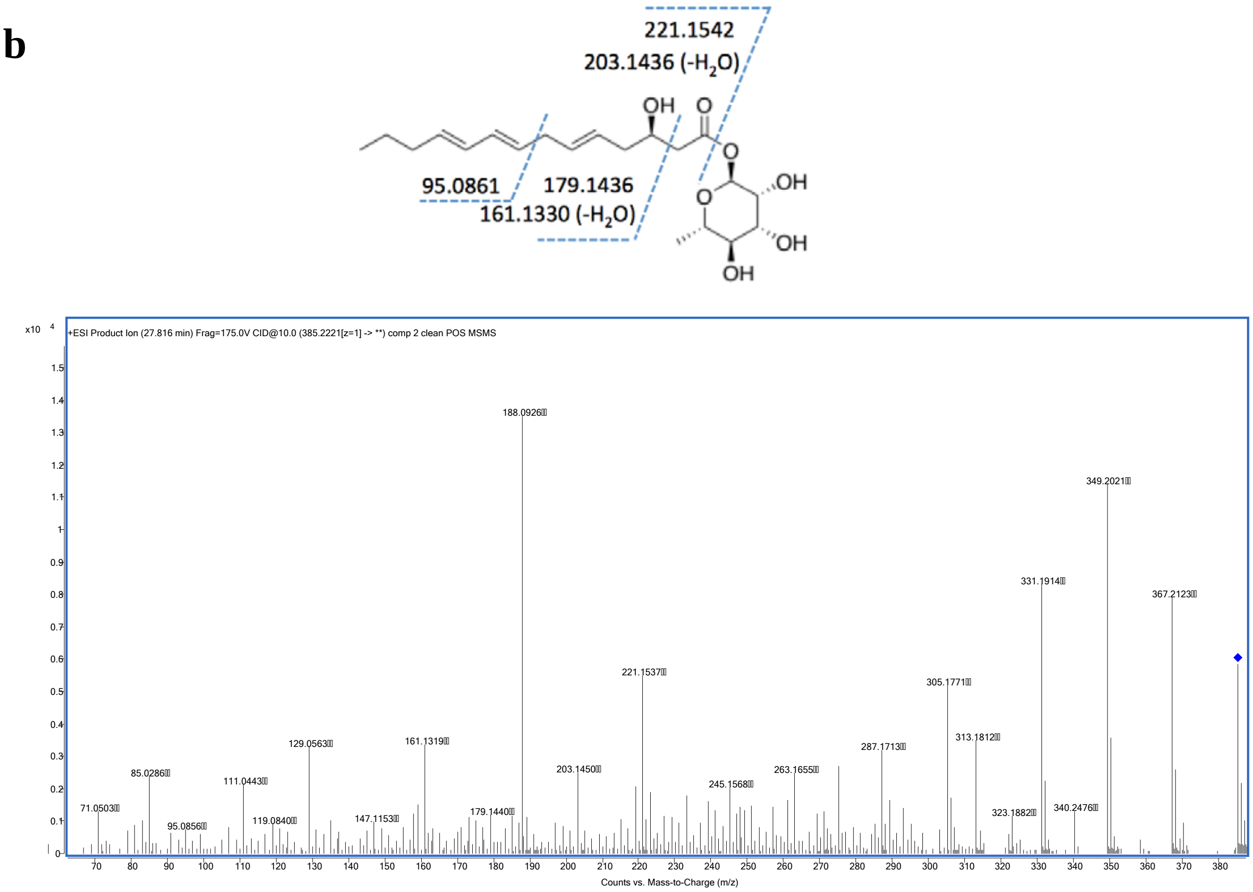
221.1542
203.1436 (-H2O)
OH
O
O
OH
OH
OH
95.0861
179.1436
161.1330 (-H2O)
x10 4
+ESI Product Ion (27.816 min) Frag=175.0V CID@10.0 (385.2221[z=1] -> **) comp 2 clean POS MSMS
188.0926
349.2021
331.1914
367.2123
221.1537
305.1771
129.0563
161.1319
287.1713
313.1812
85.0286
203.1450
263.1655
245.1568
111.0443
71.0503
179.1440
323.1882
340.2476
147.1153
119.0840
95.0856
Counts vs. Mass-to-Charge (m/z)

c

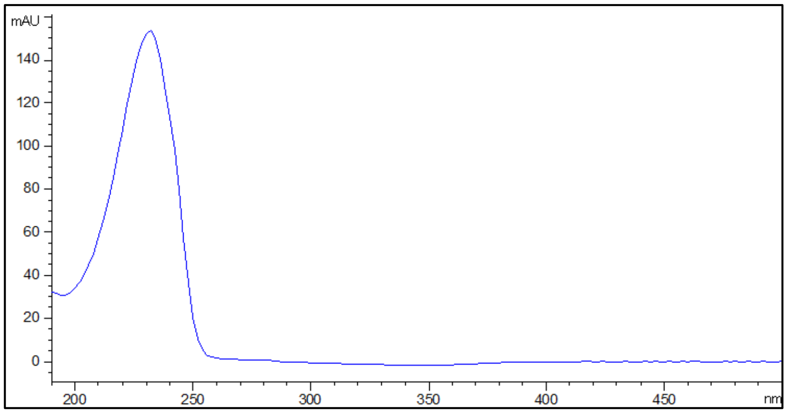

Supplementary Figure 4. HRMS and UV characterization of compound **2.** (**a**) (-)-HRMS (negative mode) of **2** (calc., 383.2075). (**b**) MS/MS analysis (positive mode) of **2**. (**c**) UV spectrum of **2**.

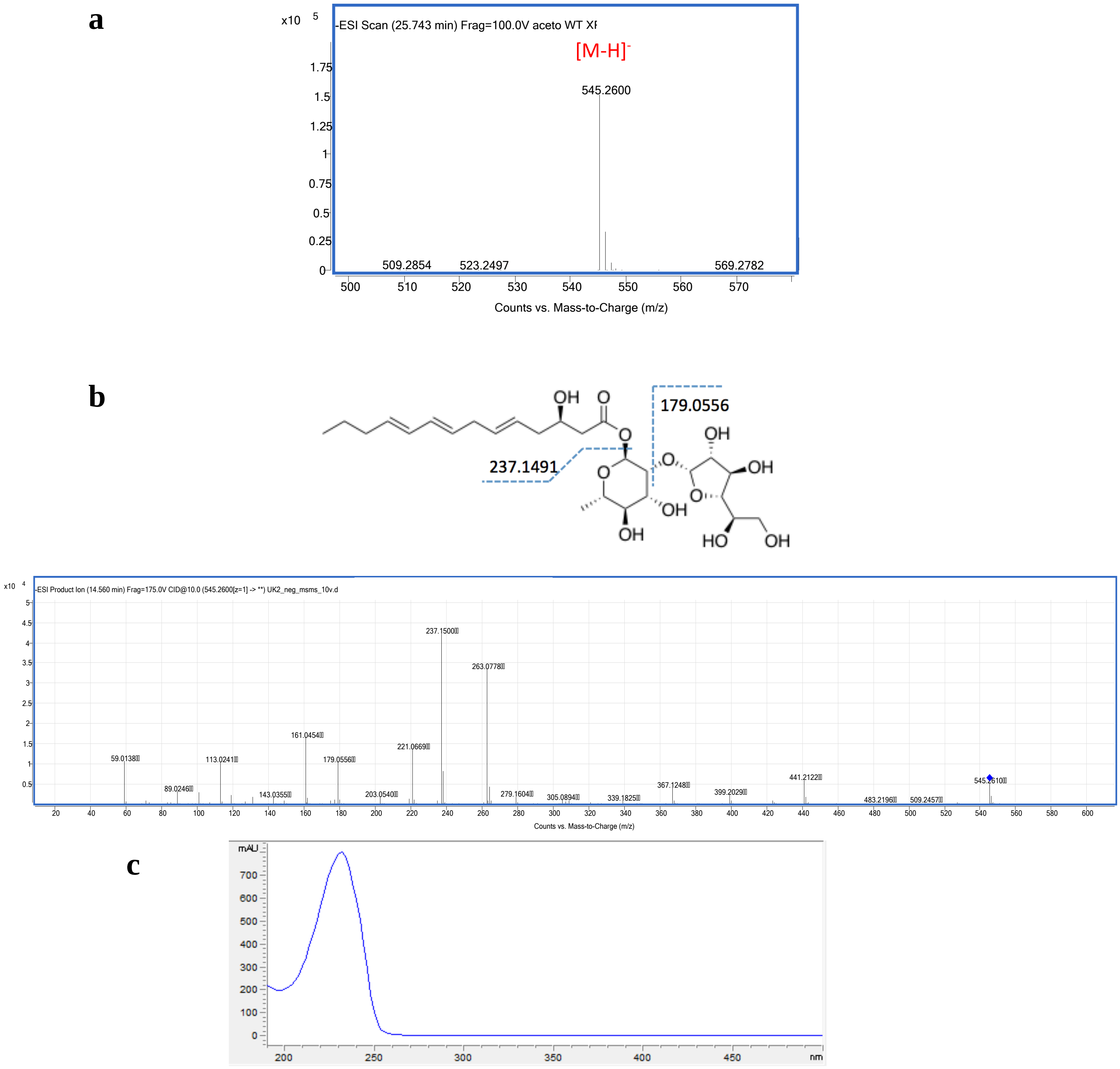

Supplementary Figure 5. HRMS and UV characterization of compound **3.** (**a**) (-)-HRMS (negative mode) of **3** (calc., 545.2604). (**b**) MS/MS analysis (negative mode) of **3**. (**c**) UV spectrum of **3**.

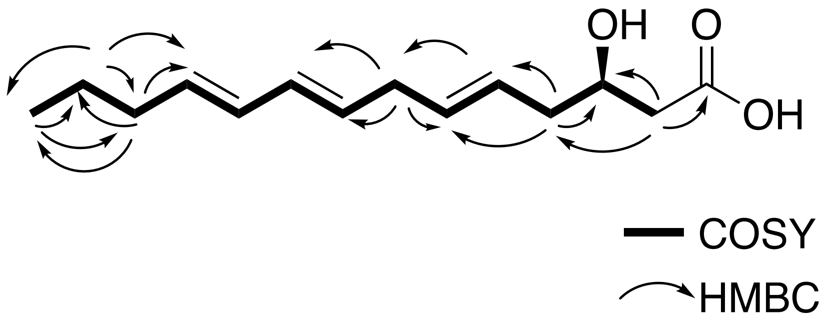

Supplementary Figure 6. NMR characterization of compound **1**. (**a**) $^{1}H,^{1}H$-COSY/TOCSY and selected HMBC correlations of **1**.

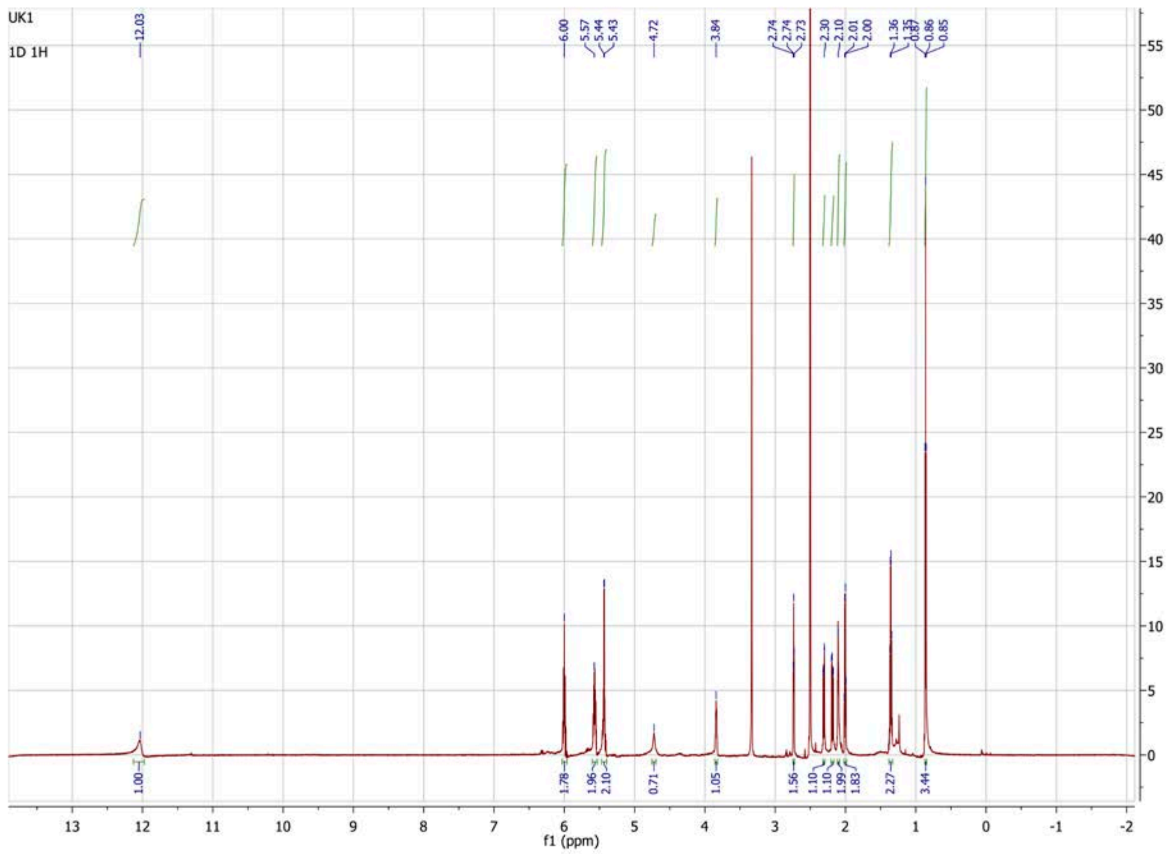

Supplementary Figure 6. (**b**) ^{1}H NMR spectrum (DMSO-d_6, 900 MHz) of **1**.

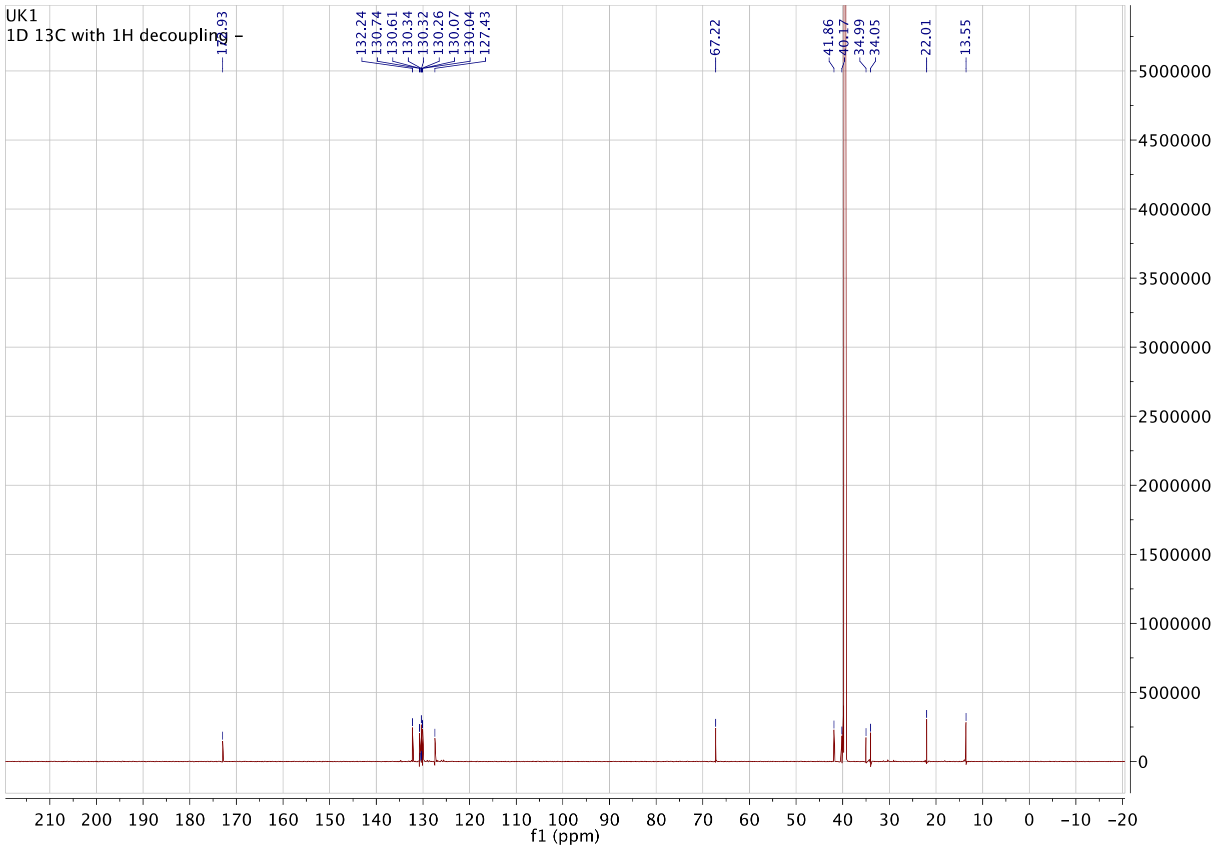

Supplementary Figure 6. (**c**) ^{13}C NMR spectrum (DMSO-d_6, 225 MHz) of **1**.

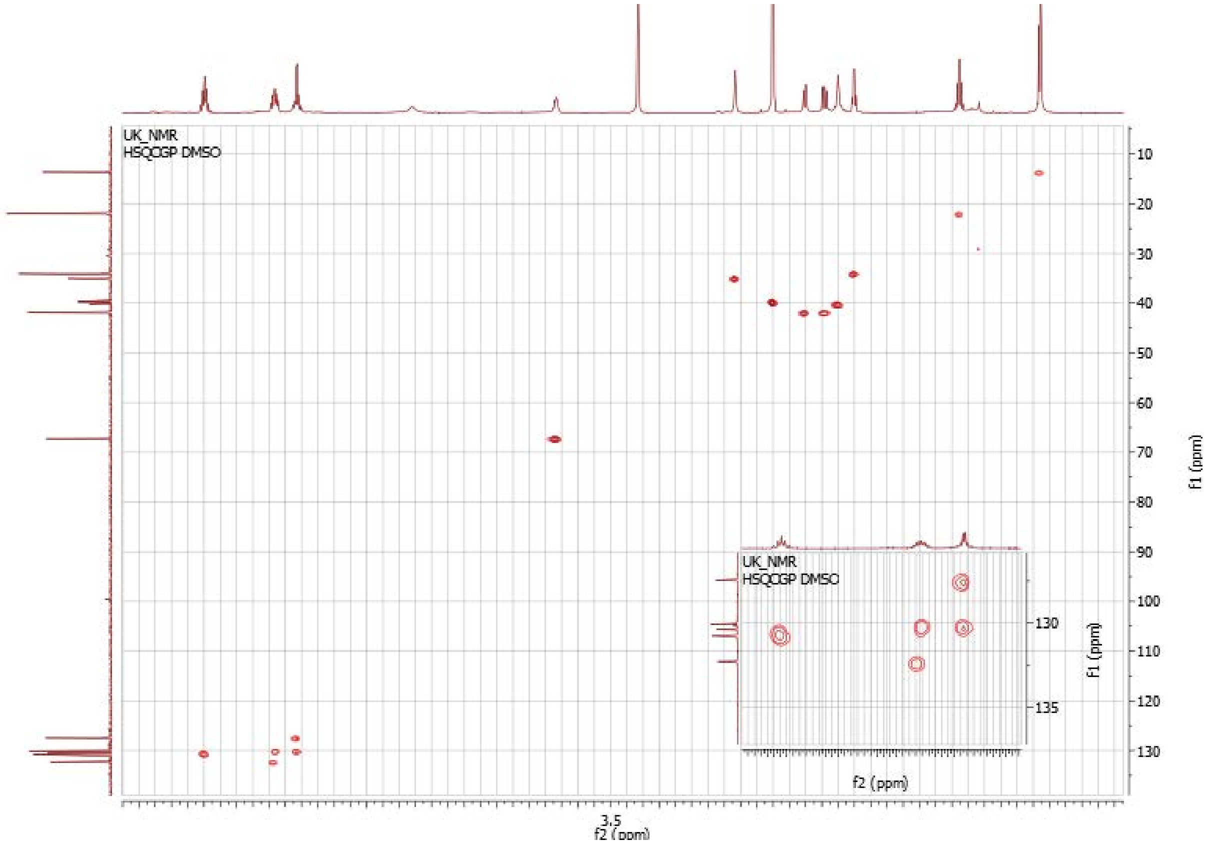

Supplementary Figure 6. (**d**) HSQC spectrum (DMSO-d_6, 900 MHz) of **1**.

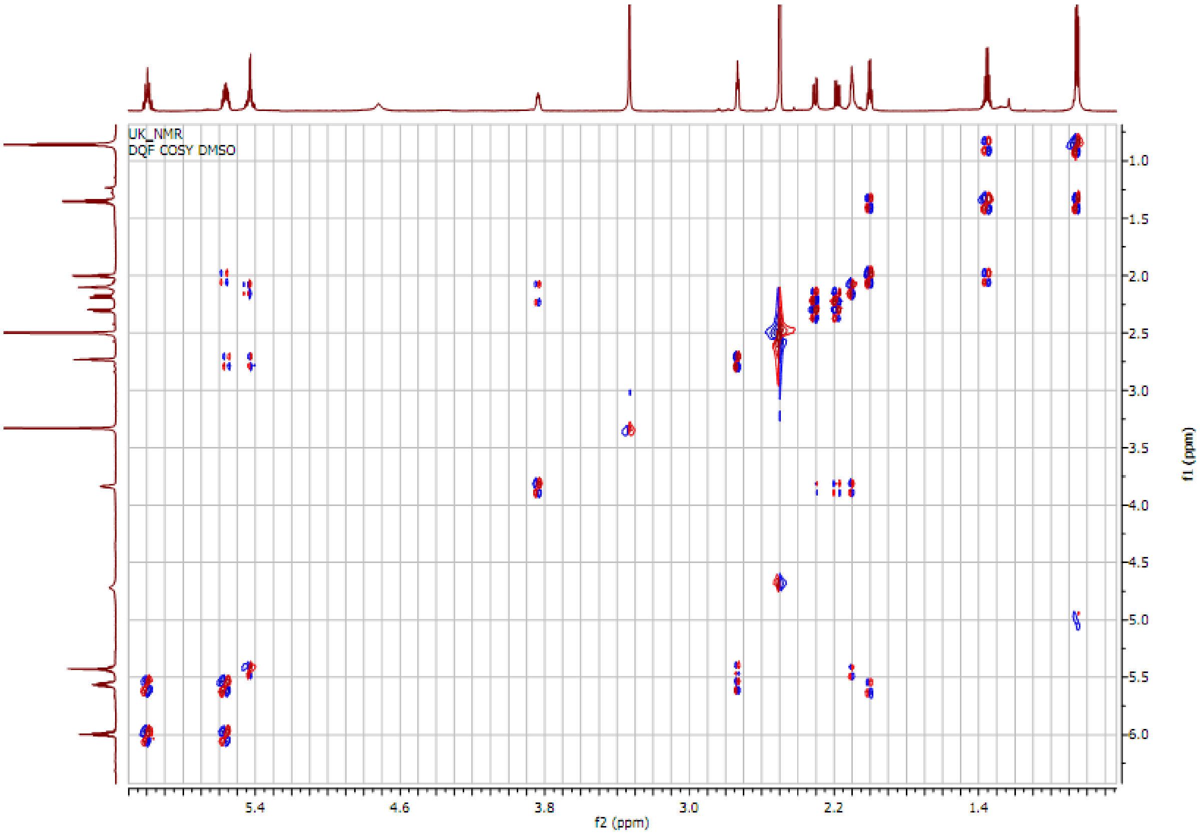

Supplementary Figure 6. (**e**) DQF-COSY spectrum (DMSO-d_6, 900 MHz) of **1**.

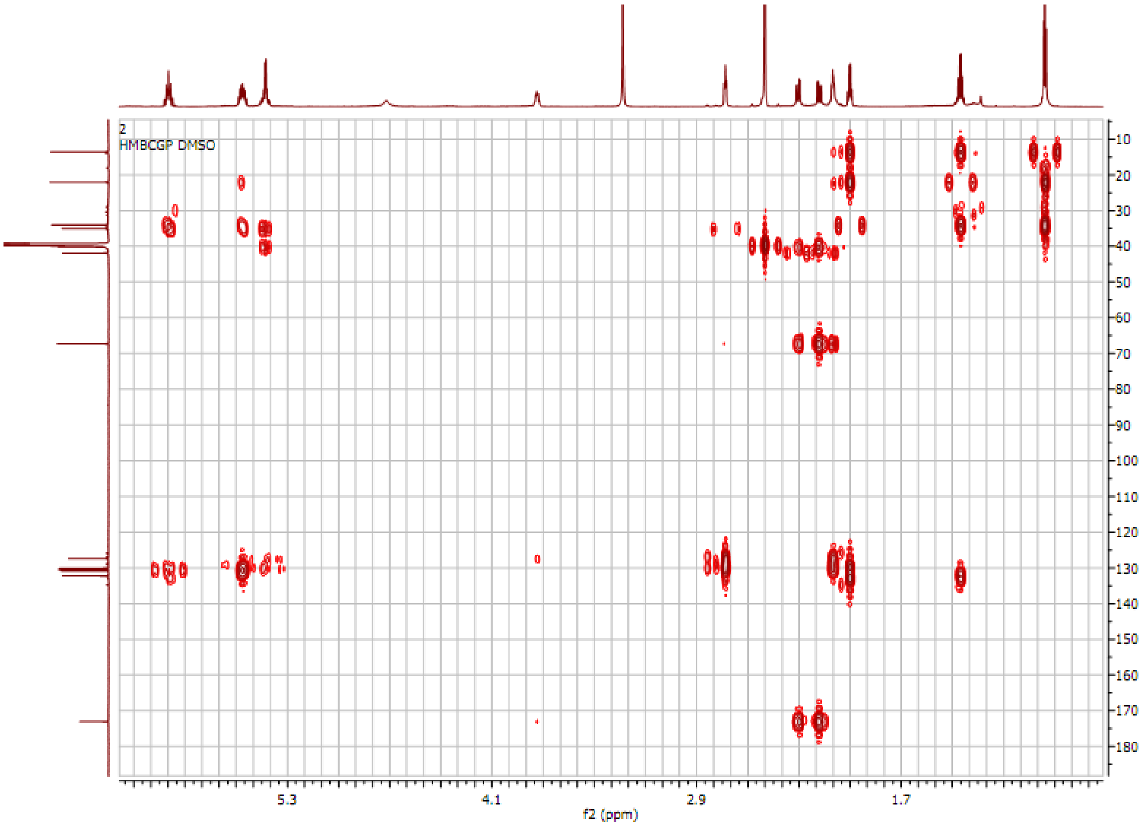

Supplementary Figure 6. (**f**) HMBC spectrum (DMSO-d_6, 900 MHz) of **1**.

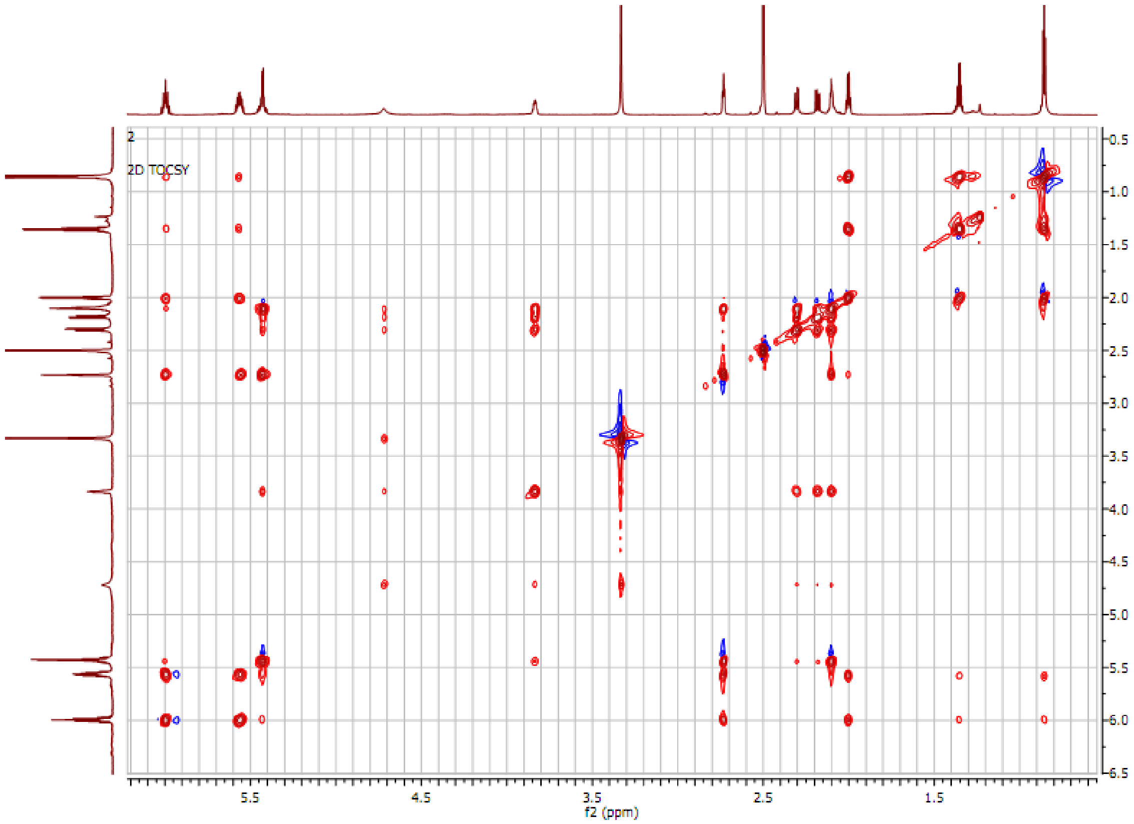

Supplementary Figure 6. (**g**) TOCSY spectrum (DMSO-d_6, 900 MHz) of **1**.

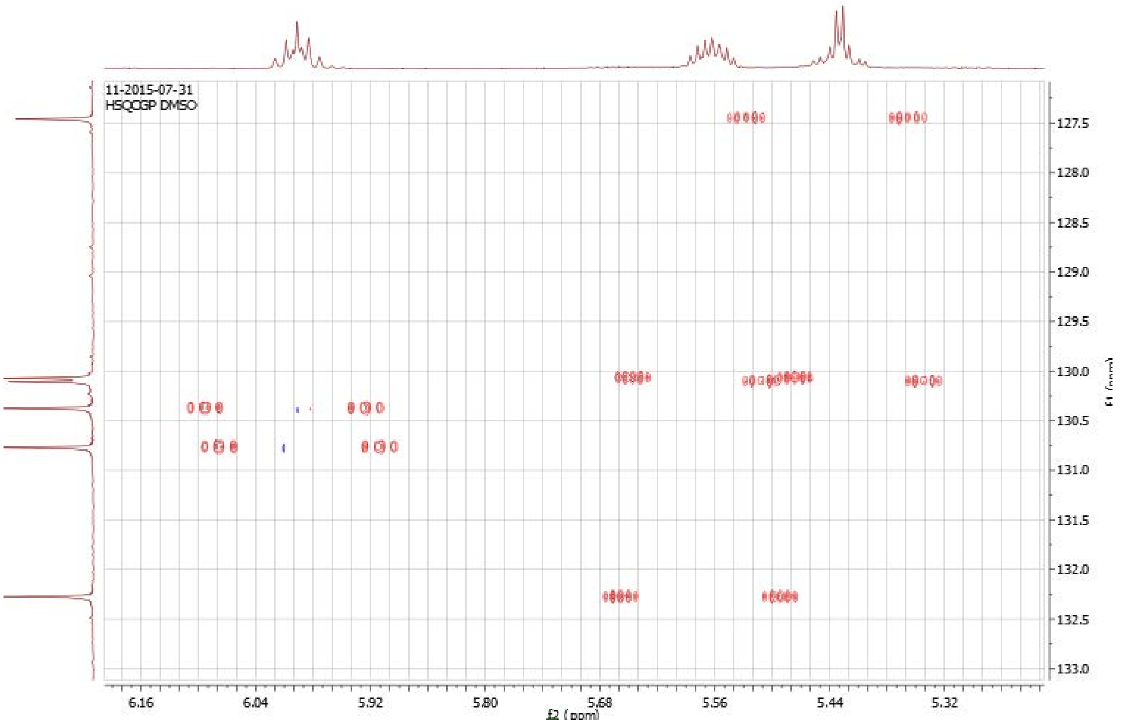

Supplementary Figure 6. (**h**) HSQC without ^{13}C decoupling spectrum (CD_3OD, 900 MHz) of **1**.
$^{3}J_{H5, H6}$ = ca 15.5 Hz; $^{3}J_{H8, H9}$ = ca 15.2 Hz; $^{3}J_{H9, H10}$ = ca 11.1 Hz; $^{3}J_{H10, H11}$= ca 15.2 Hz.

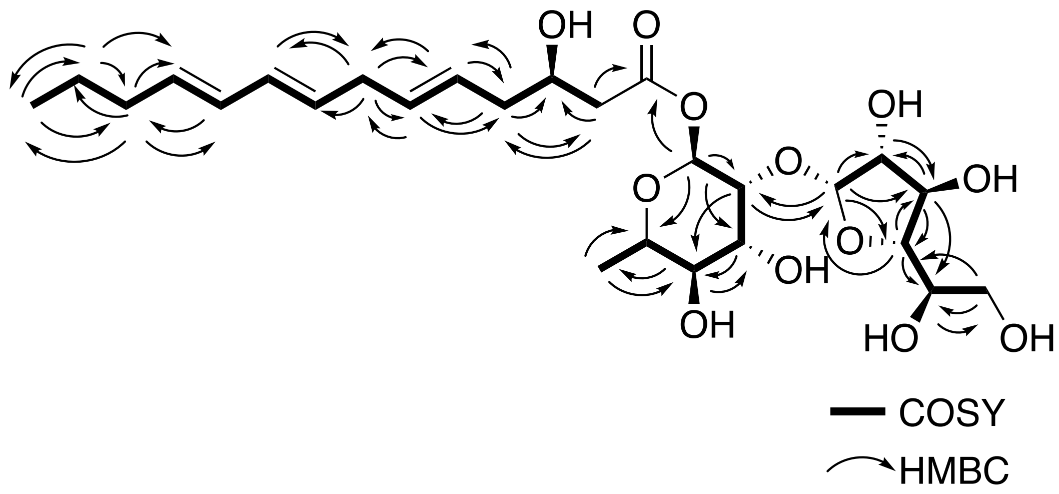

Supplementary Figure 7. NMR characterization of compound **3**. (**a**) ^{1}H,^{1}H-COSY and selected HMBC correlations of **3**.

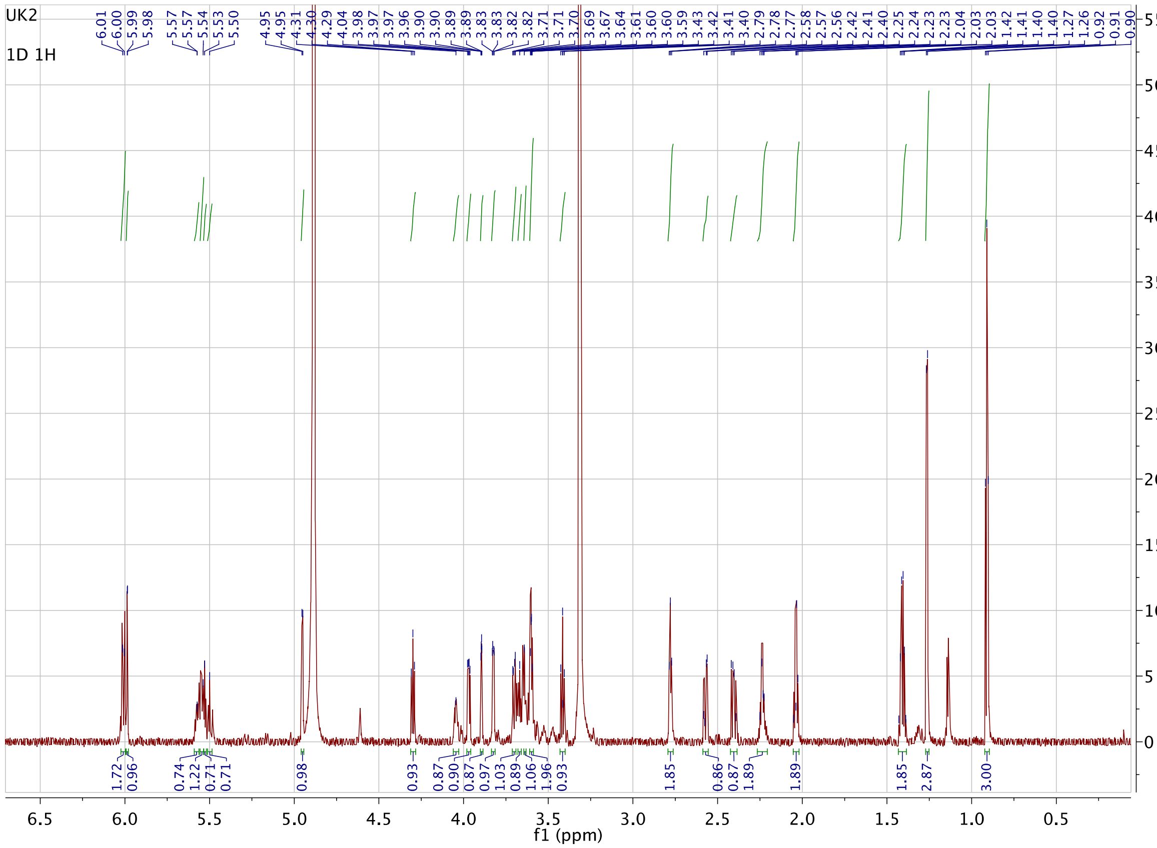

Supplementary Figure 7. (**b**) ^{1}H NMR spectrum (CD_3OD, 900 MHz) of **3**.

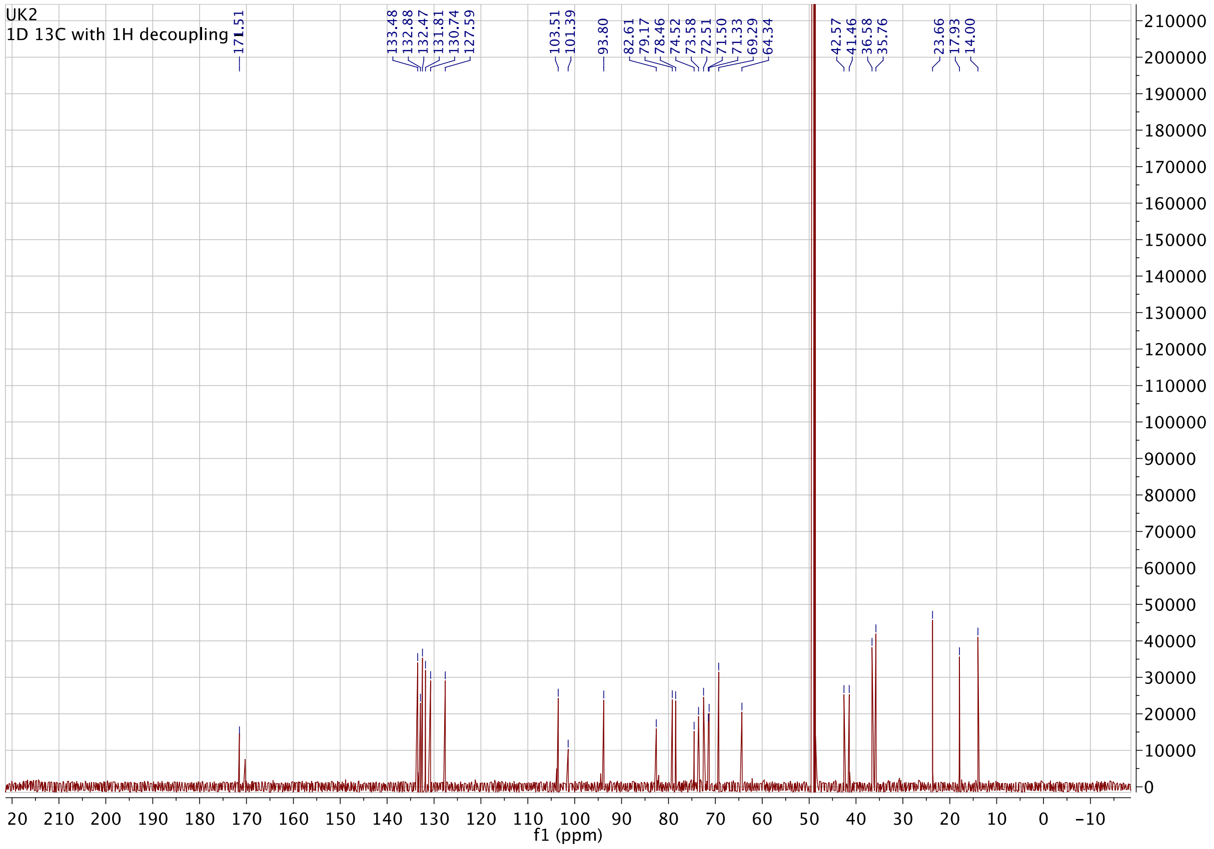

Supplementary Figure 7. (**c**) ^{13}C NMR spectrum (CD_3OD, 225 MHz) of **3**.

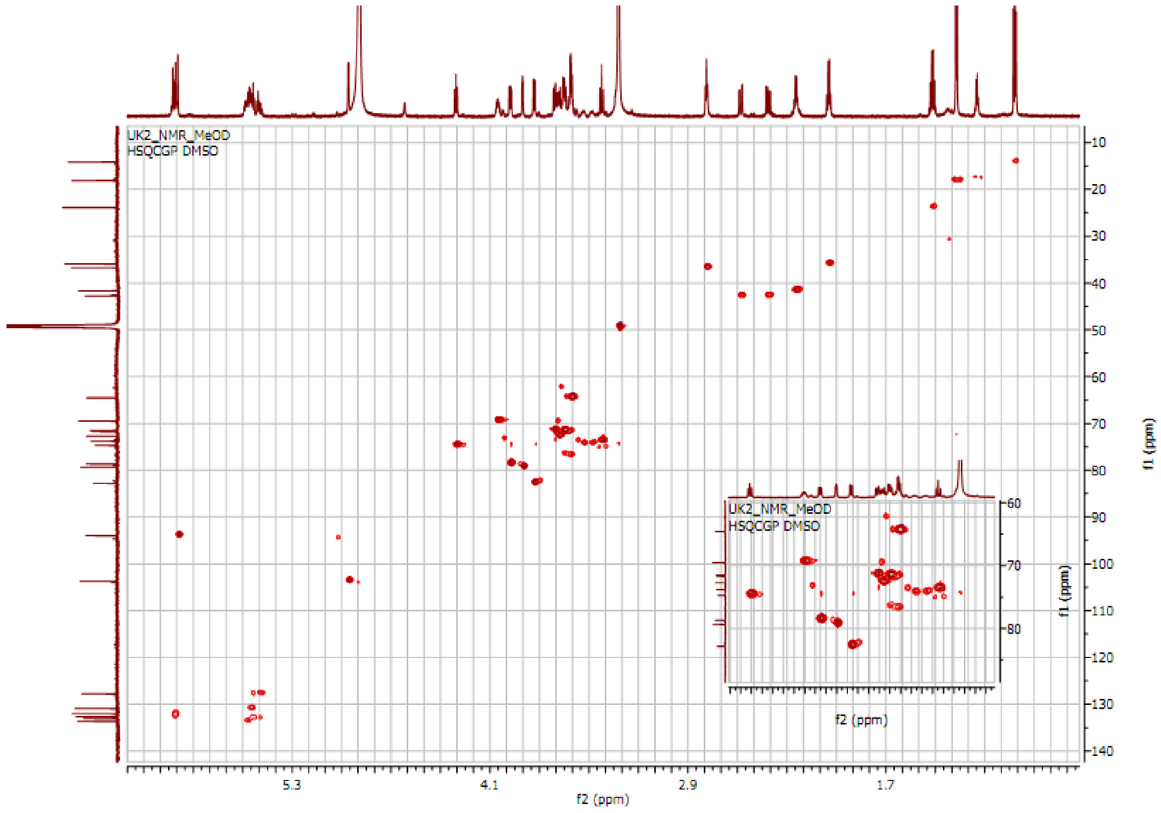

Supplementary Figure 7. (**d**) HSQC spectrum (CD_3OD, 900 MHz) of **3**.

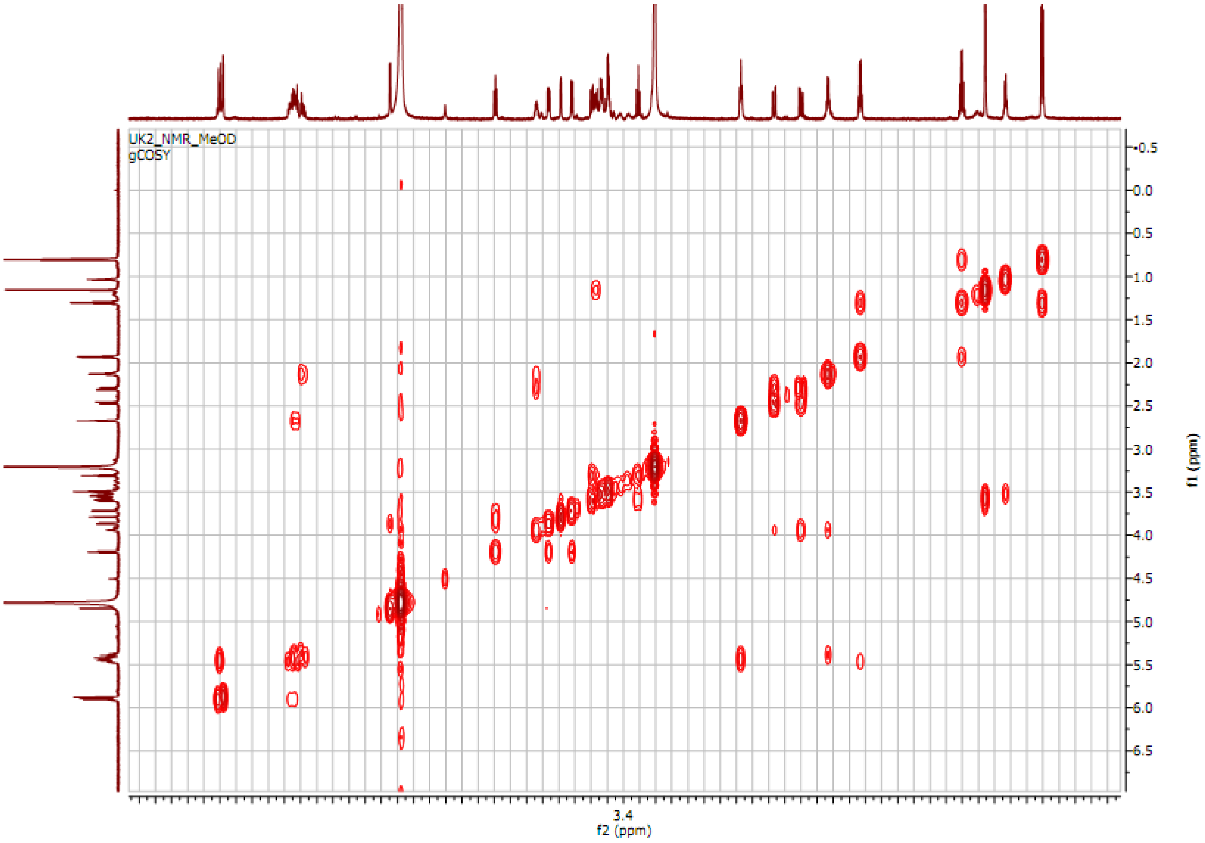

Supplementary Figure 7. (**e**) ^{1}H, ^{1}H-COSY spectrum (CD_3OD, 900 MHz) of **3**.

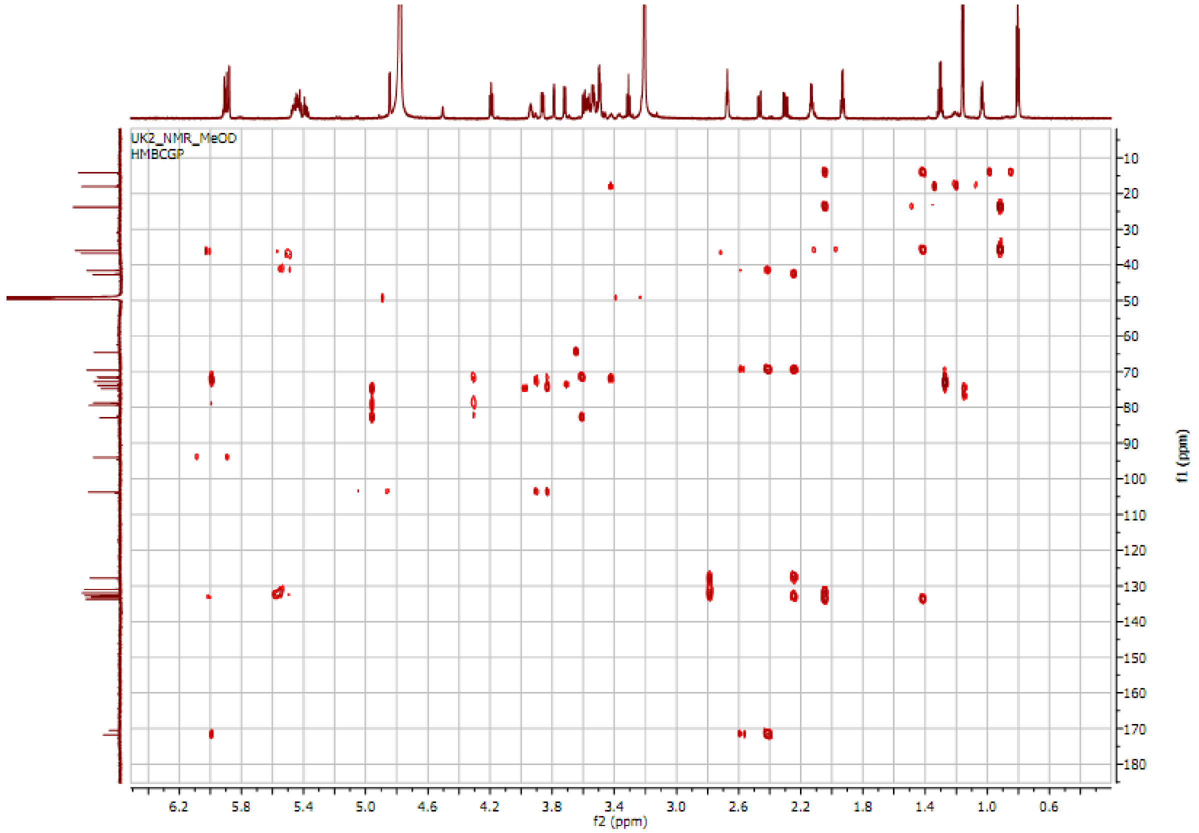

Supplementary Figure 7. (**f**) HMBC spectrum (CD_3OD, 900 MHz) of **3**.

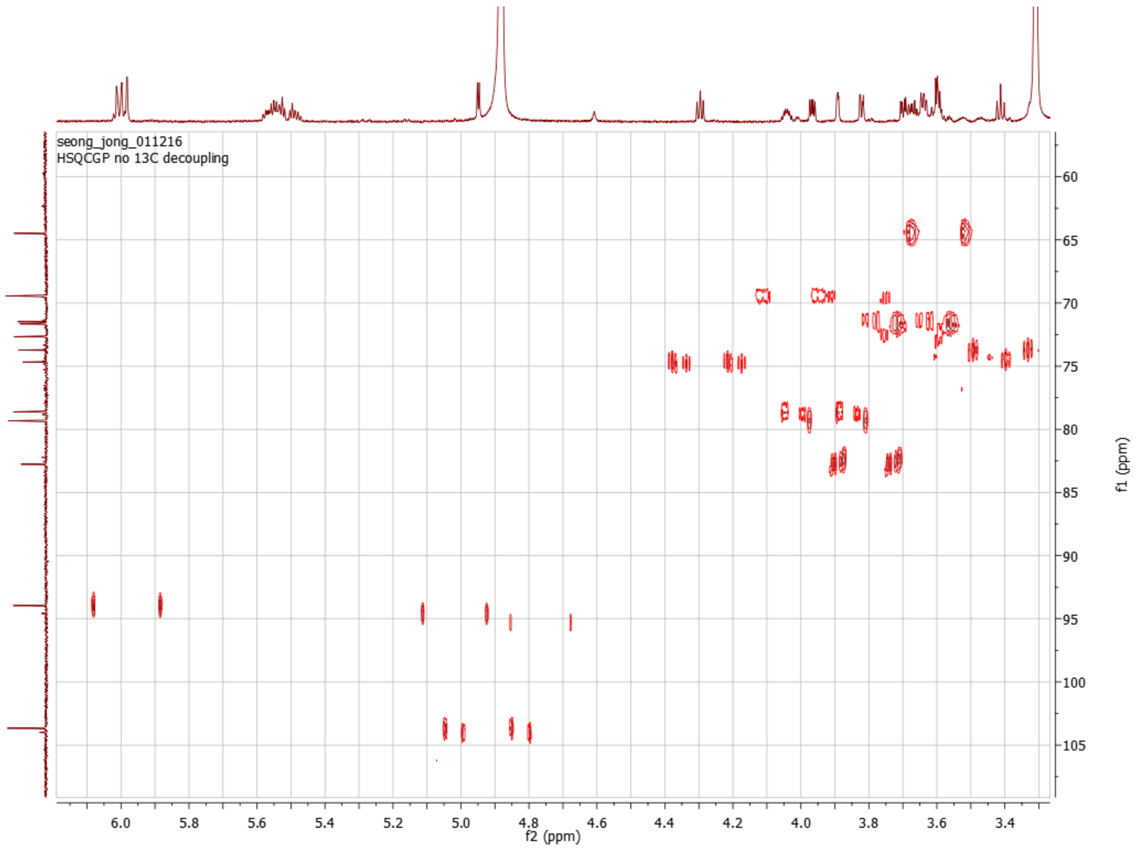

Supplementary Figure 7. (**g**) HSQC without ^{13}C decoupling spectrum (CD_3OD, 900 MHz) of **3**.

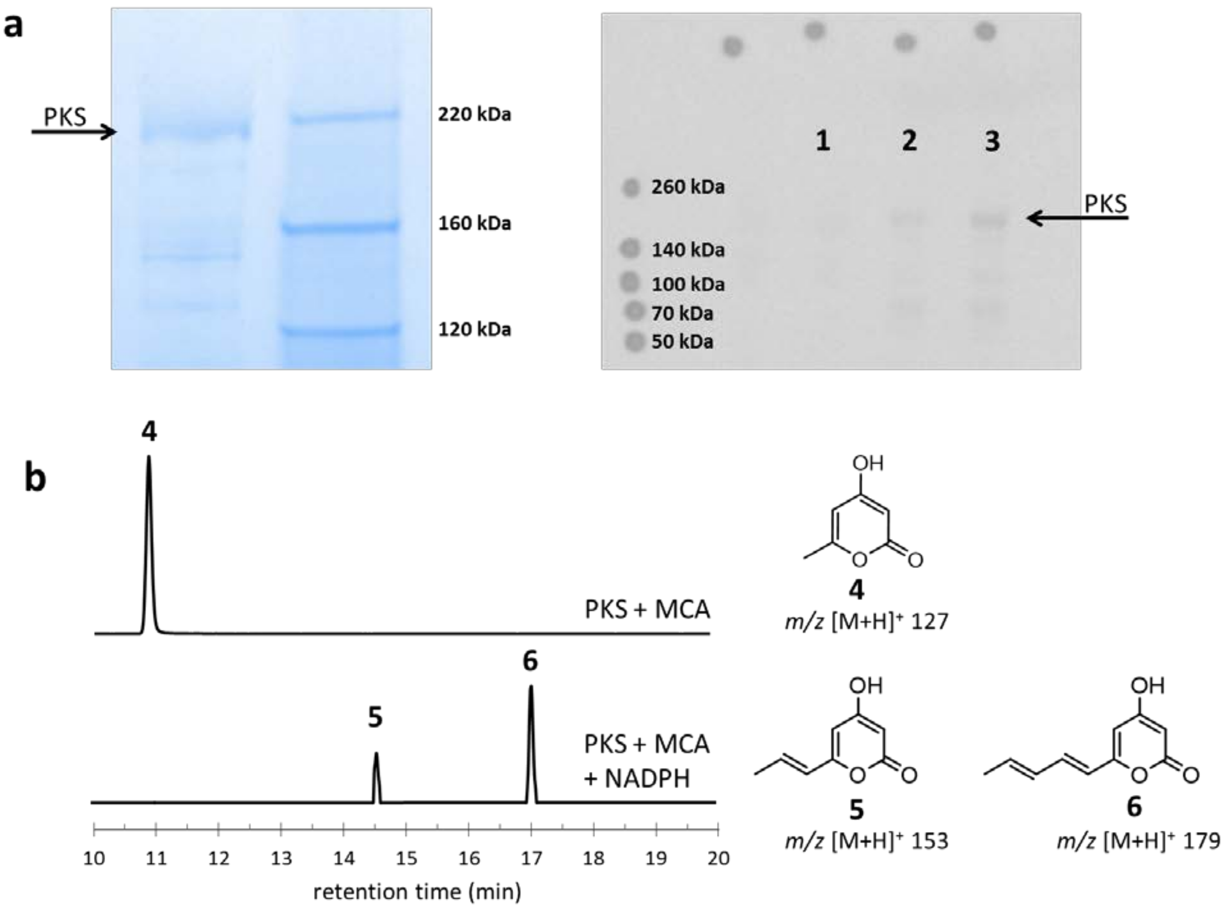

Supplementary Figure 8. *In vitro* reconstitution of PKS activity. (**a**) Left: SDS-PAGE gel showing soluble PKS protein (203 kDa) purified from *E. coli* BAP1 expressing pET24b_pks. Right: SDS-PAGE autoradiograph gel showing results of ^{14}C-labeled malonyl-CoA PKS loading assay. Lanes 2 and 3 show the results for assays performed without (Lane 2) and with (Lane 3) the addition of NADPH. Lane 1 is a negative control without PKS. (**b**) HRMS extracted ion chromatographs showing products of *in vitro* biochemical assays of purified PKS protein. Assays containing PKS and malonyl-CoA (MCA) yielded the triketide lactone compound **4** (top chromatogram), while addition of NADPH to the assay permitted the production of tetra- and penta- ketide pyrones (compounds **5** and **6**, respectively) (bottom chromatogram).

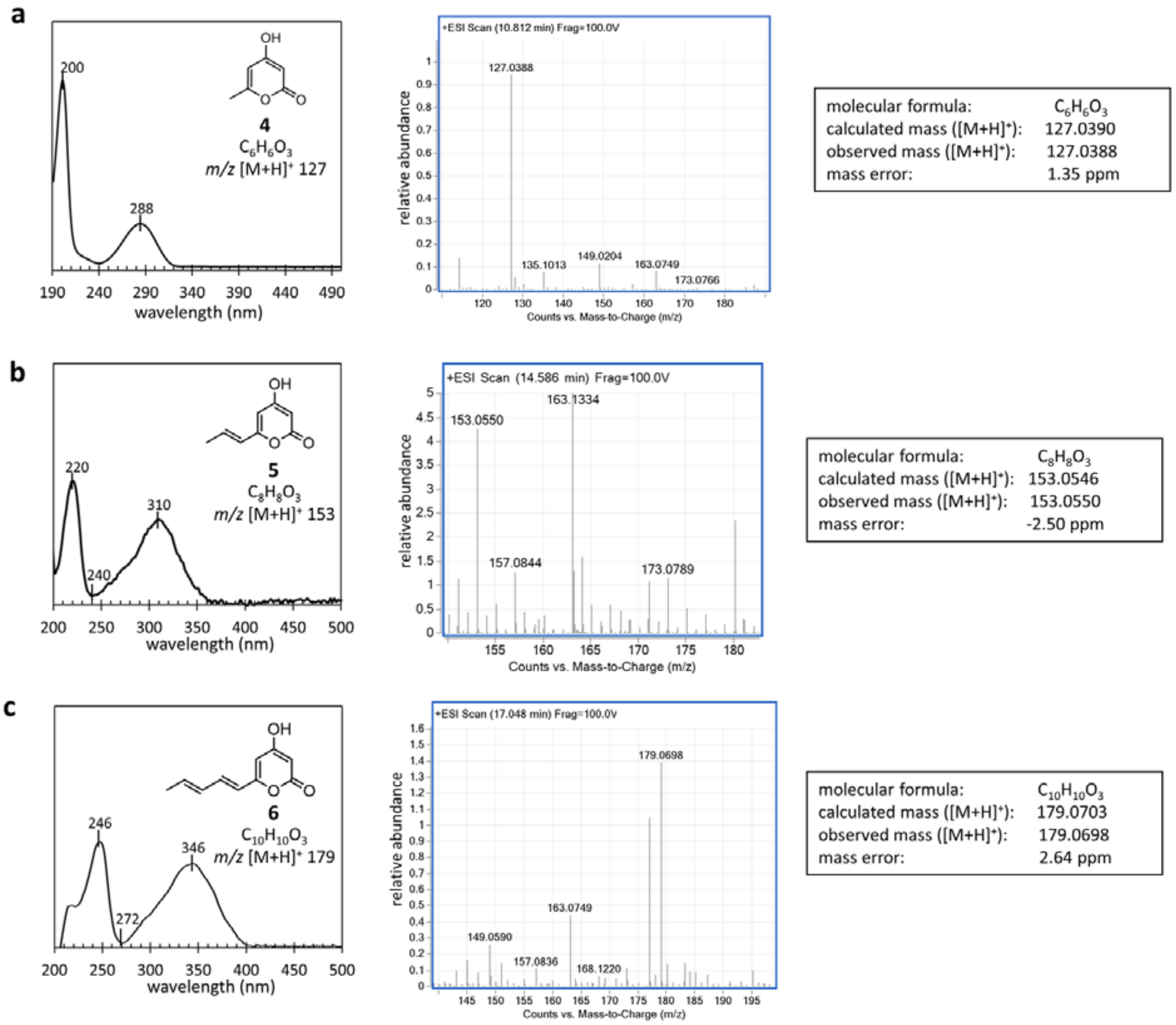

Supplementary Figure 9. HRMS and UV spectra for compounds **4**, **5**, and **6**. (**a**) Left: UV spectrum for compound **4**. Center: HRMS spectrum for compound **4**. Right: Comparison of calculated and observed masses for compound **4**. (**b**) Left: UV spectrum for compound **5**. Center: HRMS spectrum for compound **5**. Right: Comparison of calculated and observed masses for compound **5**. (**c**) Left: UV spectrum for compound **6**. Center: HRMS spectrum for compound **6**. Right: Comparison of calculated and observed masses for compound **6**. The molecular structures for **4**, **5**, and **6** were assigned based on comparison of HRMS spectra, UV spectra, and HPLC retention time to chemical standards of these compounds.

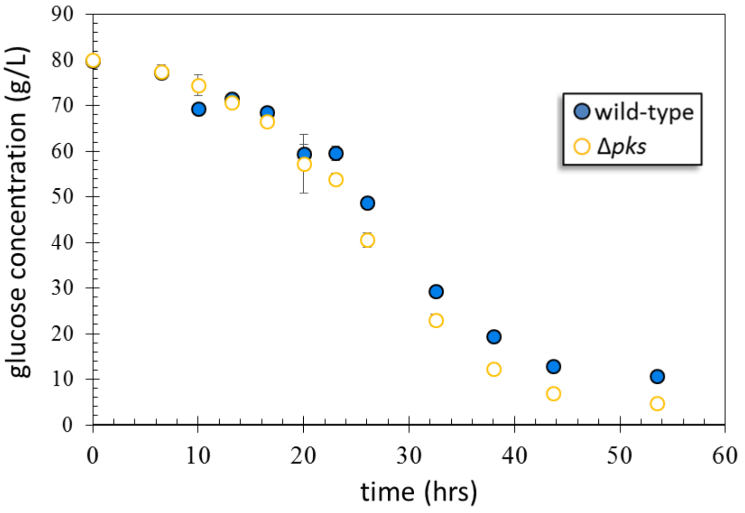

Supplementary Figure 10. Glucose concentrations for time-course batch fermentation data (complementary to **Fig. 2a-c)**. Error bars represent the standard deviation of biological duplicate experiments.

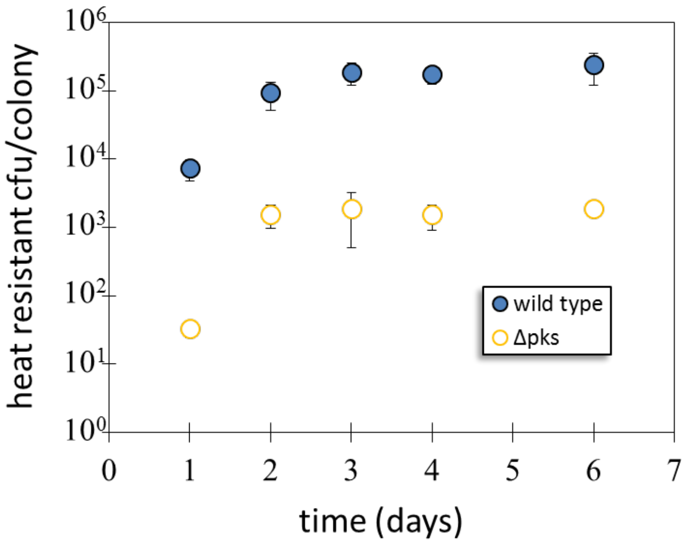

Supplementary Figure 11. Sporulation assay results (time-course) for incubation of wild-type and Δ*pks* on solid CBM medium. Error bars represent the standard deviation of biological triplicate experiments.

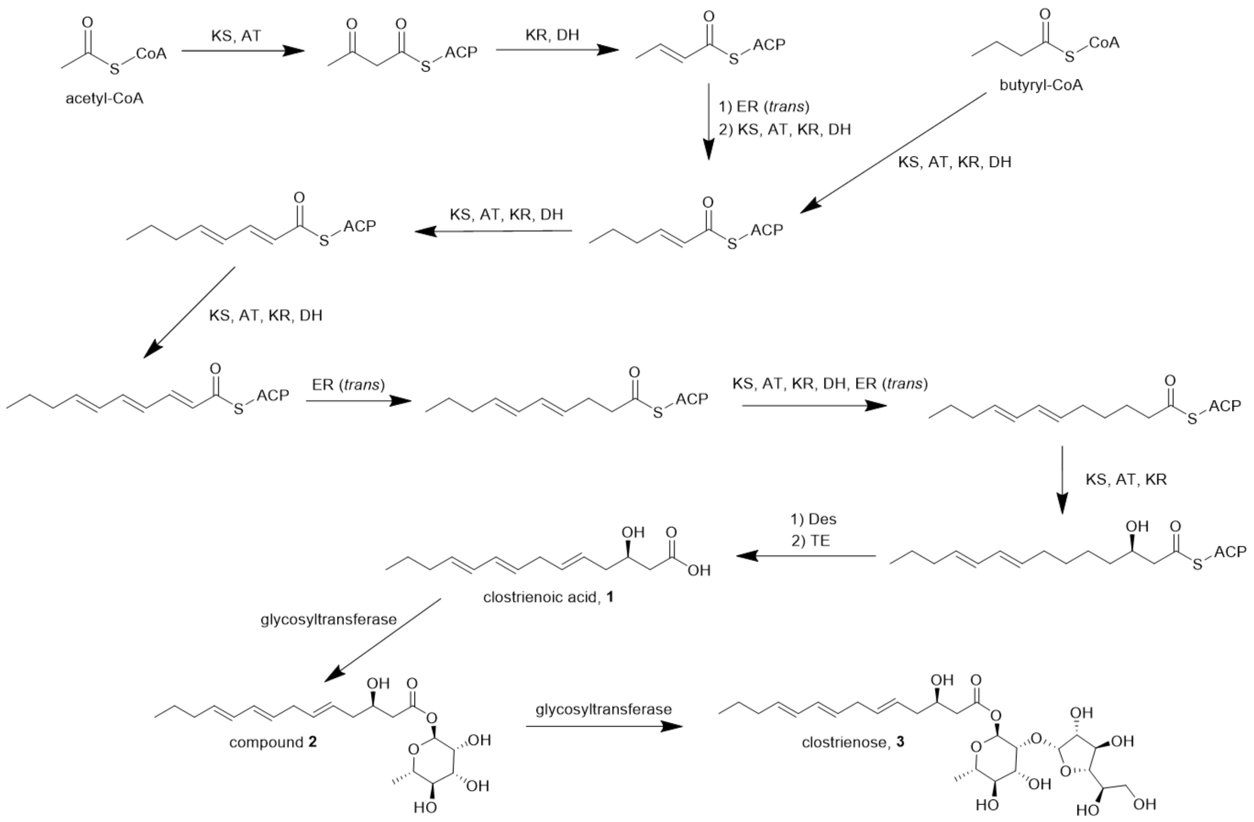

Supplementary Figure 12. Proposed biosynthetic pathway for the production of compounds **1**-**3**. Proposed PKS starter units are acetyl-CoA or butyryl-CoA. PKS domains include ACP, acyl carrier protein; KS, ketosynthase; AT, acyltransferase; KR, ketoreductase; and DH, dehydratase. Non-PKS contributors proposed are *trans*-ER, *trans*-enoyl reductase; Des, fatty-acyl desaturase; TE, thioesterase; and two glycosyltransferases. It is important to note that this proposed pathway is highly speculative given our limited knowledge of the tailoring enzymes involved. For example, an isomerase may be involved instead of ER and Des to change the position of the double bond.

Supplementary Table 1. ^{1}H (900 MHz), ^{13}C (226 MHz) ^{1}H,^{1}H-COSY and HMBC NMR data for clostrienoic acid (**1**).

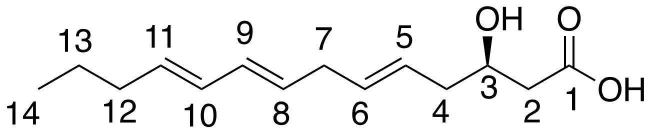

No.	δ_C	δ_H	Type	COSY	HMBC
1	172.94	12.03, br. s	COOH		
		2.18, dd (15.0, 8.3)			
		2.31, dd (15.0, 4.7)			
3	67.23	3.84, m	CH	2, 4, 3-OH	1, 5
3-OH		4.72, br. s	OH	3	
4	40.17	2.10, m	CH_2	3, 5	3, 5, 6
5	127.43	5.44, m	CH	4, 6	5, 7
6	130.08	5.42, m	CH	5, 7	4, 7
7	35.00	2.73, t (5.7)	CH_2	6, 8	5, 6, 8, 9
8	130.04	5.56, m	CH	7, 9	
9	130.74	6.00, m	CH	8, 10	7, 11
10	130.35	6.00, m	CH	9, 11	8, 12
11	132.25	5.57, m	CH	3, 5	9, 12
12	34.05	2.00, dt (7.2, 7.2)	CH_2	11, 13	10, 11, 13, 14
13	22.02	1.35, tq (7.2, 7.7)	CH_2	12, 14	11, 12, 14
14	13.55	0.86, t (7.7)	CH_3	13	12, 13

Supplementary Table 2. ^{1}H (900 MHz), ^{13}C (226 MHz) ^{1}H,^{1}H-COSY and HMBC NMR data for clostrienose (**3**).

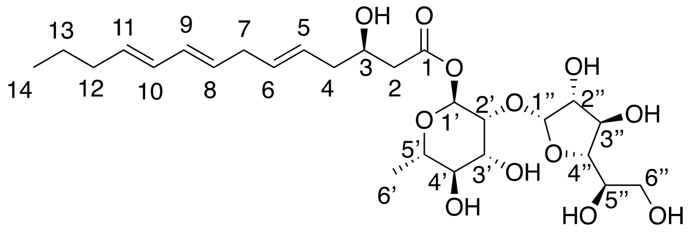

No.	δ_C	δ_H	Type	COSY	HMBC
1	171.51		C		
2	42.57	2.40, dd (15.3, 8.8)<br>2.57, dd (15.3, 4.0)	CH_2	3	1, 3, 4
3	69.29	4.04, m	CH	2, 4	
3-OH			OH		
4	41.45	2.23, m	CH_2	3, 5	2, 3, 5, 6
5	127.59	5.49, br. dt (15.4, 6.6)	CH	4, 6	4, 7
6	132.88	5.53, br. dt (15.4, 6.6)	CH	5, 7	4, 7
7	36.58	2.78, br. t (6.2)	CH_2	6, 8	5, 6, 8, 9
8	130.74	5.54, m	CH	7, 9	
9	132.47	6.01, m	CH	8, 10	7
10	131.82	6.01, m	CH	9, 11	12
11	133.48	5.57, m	CH	10, 12	
12	35.77	2.03, br. dt (7.0, 7.3)	CH_2	11, 13	10, 11, 13, 14
13	23.66	1.41, tq (7.3, 7.4)	CH_2	12, 14	11, 12, 14
14	14.00	0.91, t (7.4)	CH_3	13	12, 13
1’	93.80	5.98, d (1.6)	CH	2’	1, 2’, 3’, 5’
2’	79.17	3.89, dd (3.7, 1.6)	CH	1’, 3’	4’, 1’’
3’	71.33	3.70, dd (9.6, 3.6)	CH	2’, 4’	4’
4’	73.58	3.41, dd (9.8, 9.6)	CH	3’, 4’	3’, 6’
5’	72.52	3.67, dq (9.8, 6.2)	CH	4’, 6’	
6’	17.94	1.26, d (6.2)	CH_3	5’	4’, 5’
1’’	103.51	4.95, d (4.8)	CH	2’’	2’, 2’’, 3’’, 4’’
2’’	78.46	3.97, dd (8.6, 4.8)	CH	1’’, 3’’	3’’
3’’	74.52	4.30, dd (8.6, 7.7)	CH	2’’, 4’’	2’’, 4’’, 5’’
4’’	82.61	3.82, dd (7.7, 1.7)	CH	3’’, 5’’	1’’, 3’’
5’’	71.51	3.64, ddd (7.1, 5.9, 1.7)	CH	4’’	6’’
6’’	64.34	3.60, dd (10.8, 5.9)<br>3.59, dd (10.8, 7.1)	CH_2	5’’	4’’, 5’’

Supplementary Table 3. Oil spreading assay results[a] for surfactin (positive control), purified clostrienose (**3**), and 10 mM potassium phosphate buffer negative control.

	10 µM<br>pH 5.0	10 µM<br>pH 8.0	100 µM<br>pH 5.0	100 µM<br>pH 8.0
surfactin	++++	++++	+++++	+++++
clostrienose	o	+	++	+++
control	o	o	o	o

[a]o, no oil film disturbance; +, unstable clearing zone < 5 mm diameter; ++, stable clearing zone 5 – 10 mm diameter; +++, stable clearing zone 10 – 15 mm diameter; ++++, near complete oil film dispersal; +++++, complete oil film dispersal. Solutions were prepared in 10 mM potassium phosphate buffer at the indicated pH and concentration.

Supplementary Table 4. Drop collapse assay results[a] for surfactin (positive control), purified clostrienose (**3**), and 10 mM potassium phosphate buffer negative control.

	10 µM	10 µM	100 µM	100 µM
	pH 5.0	pH 8.0	pH 5.0	pH 8.0
surfactin	+++	+++	++++	++++
clostrienose	o	o	o	o
control	o	o	o	o

[a]o, stable droplet bead ~ 2 mm diameter; +++, collapsed droplet ~ 4 mm diameter; ++++, collapsed droplet ~ 6 mm diameter. Solutions were prepared in 10 mM potassium phosphate buffer at the indicated pH and concentration.

Supplementary Table 5. Primers used in this study.

Primer	Sequence (5' to 3')[a]
PKS_Fo	AAAGAATTCAATGGCTAAAATAGCTATAATAGG
PKS_Ro	AAACTCGAGTGCAGTTACTTTGCTCATCA
pKON_Fo	AAAAGACGATAGTCCTGTGGATGGAGTTAAGTCAG
pKON_Ro	AAAAGACTATCGTCGTAATACGGTTATCCACAGAATCAG
UHR_Fo	TGCTCACATGTTCTTTCCTGCACTACTGTTTGTGCTTGCAG
UHR_Ro	CACAGAATCAGGGGATAACGGCCTGGAAGCCTTAATCC
DHR_Fo	TAAACCTGCACCGGTCCTAGGCTGATGTAATGATGAGCAA
DHR_Ro	AGTATTCGTCAATCGGGATCCAAAGGATTAACCGCTCC
CMR_F	CGTTATCCCCTGATTCTGTG
CMR_R	CTAGGACCGGTGCAGGTT
pKO_F	GATCCCGATTGACGAATACTA
pKO_R	CAGGAAAGAACATGTGAGC
pAN1_F	GCTCCTGAAAATCTCGATAACT
pAN1_R	GCAGTTATTGGTGCCCTTAA
KAN_Fo	TTAAGGGCACCAATAACTGCGTCTCCGCAAGTGGCACT
KAN_Ro	TTATCGAGATTTTCAGGAGCGCATGCCTATTTGTTTATTTTTC
CPKS_Fo	CATTATTAAATTTAAGGAGGAACATATCATATGGCTAAAATAGCTATAATAGGCTTGG
CPKS_Ro	CGGGCAGTTTAGGCGGGTTTAAACTTATGCAGTTACTTTGCTCATC
pWIS_F	GTTTAAACCCGCCTAAACTG
pWIS_R	CATATGATATGTTCCTCCTTAAATTTAATAATG
pCKO_F	CAAGAATATCACACAAAGATAAACAG
pCKO_R = P2	CATATGACAATCTGTTTGAAACC
PKS_US_F = P1	GTTTCAGCTAAGTCGTTGCTAG
PKS_DS_R = P3	CAAGCATGGAACCTCATAAG
THI_F = P5	GCAACGGTATGGAAACAAT
THI_R = P4	CGGTCATGCTGTATGTACAA

[a]Underlined portions represent overhang sequences added for Gibson assembly or restriction digest cloning.

Supplementary Methods

Purification and structure elucidation of compounds 1-3. To yield sufficient amounts of compounds **1**, **2**, and **3** for complete structure elucidation, 34 L of wild-type *C. acetobutylicum* culture was generated from several rounds of fermentation using a New Brunswick Scientific Bioflow 115 Benchtop Fermenter. Overnight cultures (10 mL CGM, 30 g/L glucose, stagnant, 37°C) inoculated with heat shocked individual colonies of *C. acetobutylicum* were cultured until reaching OD_{600} ~ 1. A 10% inoculum was then used to start a subculture (50 mL CGM, 30 g/L glucose, stagnant, 37°C), and the subculture was incubated until reaching OD_{600} ~ 1. A 10% inoculum was used to start the final subculture (300 mL CGM, 80 g/L glucose, stagnant, 37°C), and the final subculture was incubated until reaching OD_{600} ~ 1. The 300 mL culture was then aseptically transferred to the Bioflow 115 fermenter pre-loaded with 10.0 L of nitrogen-sparged CGM (80 g/L glucose, 1 mL Antifoam 204, 37°C). The fermentation was then allowed to proceed for 72 hours. The temperature was maintained at 37°C, agitation was provided by stirring at 200 rpm, and the pH was maintained above 5.0 via automatic addition of 3 M NH_4OH.

After combining the pooled fermentation culture (34 L) and removing the cell pellets via centrifugation (3,500 xg, 15 min, room temperature), the cell-free culture supernatant was extracted using two volumes of ethyl acetate. Following isolation of the organic extract, the solvent was removed by rotary evaporation and the residue was redissolved in dichloromethane. The dark yellow oily residue (10.8 g) was subjected to silica gel column chromatography (60 Å, 220-440 mesh), and the compound-rich fractions were eluted with an ethyl acetate/hexane gradient system. Each of the compound-rich fractions were combined and concentrated to dryness (3.6 g of compound **1**, 1.1 g of compound **2**, and 2.4 g of compound **3**). Further purification of compounds **1**, **2** and **3** was performed individually. Each yellow residue of compounds **1**, **2** and **3** was subjected to reverse-phase high-performance liquid chromatography (RP-HPLC, Agilent 1260 HPLC with

DAD) on a semi-preparative C18 column (10 µm, 250 x 22 mm, 300 Å, Vydac) with a linear gradient of 2-98% CH_3CN (vol/vol) over 1 hour in H_2O with 0.1% formic acid at a flow rate of 5 mL/min. Product-rich fractions containing compound **1**, **2** and **3**, respectively, were collected manually and concentrated under vacuum. The residue was redissolved in methanol (2-4 mL), and was again purified by RP-HPLC (Agilent 1260 HPLC with DAD) using a C18 column (5 µm, 250 x 10 mm, 300 Å, Vydac) with a linear gradient of 25-80% CH_3CN (vol/vol) over 30 min in H_2O with 0.1% formic acid (vol/vol) at a flow rate of 2 mL/min. Fractions containing compound **1**, **2** and **3**, respectively, were collected manually and concentrated under vacuum. The residues of compound **1**, **2** and **3** were redissolved in methanol (0.5-2 mL) for further purification. Purification was again performed by RP-HPLC using a C18 column (5 µm, 250 x 4.6 mm, 100 Å, Inertsil ODS-4 column) with an isocratic system of 22% (compound **3**), 25% (compound **2**), and 30% (compound **1**) CH_3CN (vol/vol) over 30 min in H_2O with 0.1% formic acid (vol/vol) at a flow rate of 1 mL/min. Fractions containing compound **1**, **2** and **3**, respectively, were collected manually and concentrated under vacuum. The resulting purified compounds **3** were dried (0.9 mg) and analyzed by HRMS and NMR. The amount of **2** was too low for NMR analysis. The residue of compound **1** required further purification, and was redissolved in methanol (0.5 mL) again for the final purification. The final purification was performed by RP-HPLC using a C18 column (3.5 µm, 100 x 4.6 mm, Agilent Eclipse Plus) with an isocratic system of 26% CH_3CN (vol/vol) over 30 min in H_2O with 0.1% formic acid (vol/vol) at a flow rate of 0.5 mL/min. Fractions containing compound **1** were collected manually and concentrated under vacuum. The resulting purified compound **1** was dried (1.1 mg) and analyzed by HRMS and NMR. LC-HRMS analysis was performed on an Agilent Technologies 6520 Accurate-Mass QTOF LC-MS instrument fitted with an Agilent Eclipse Plus C18 column (4.6 x 100 mm). A linear gradient of 2-98% CH_3CN (vol/vol)

over 40 min in H_2O with 0.1% formic acid (vol/vol) at a flow rate of 0.5 mL/min was used. NMR spectra were recorded on a Bruker Biospin 900 MHz spectrometer with a cryoprobe in d4-methanol or d6-DMSO (Cambridge Isotope Laboratories).

To determine the absolute stereochemistry of the secondary alcohol in **1**, an additional 15 mg of **1** was purified and dissolved in 10 ml of absolute EtOH, and 0.75 mg of 10% Pd/C was added. The flask was purged with hydrogen, and the reaction was stirred at room temperature for 3 hours under positive pressure of hydrogen. Reaction progress was monitored by TLC. The reaction was quenched by filtration, and the solvent was dried under vacuum to yield 3-hydroxytetradecanoic acid (3-HTA) as a white powder (reaction yield: 75%). The resulting 3-HTA product was analyzed by LC-HRMS, ^{1}H NMR, ^{13}C NMR, and the specific rotation was measured. The specific rotation of **1** was also measured.

Compound 1 Data:

^{1}H NMR (900 MHz, DMSO-d_6) δ 12.02 (s, 1H), 6.00 (m, 2H), 5.57 (m, 1H), 5.56 (m, 1H), 5.44 (m, 1H), 5.42 (m, 1H),4.72 (s, 1H),3.84 (m, 1H), 2.73 (t, J = 5.7 Hz, 2H), 2.31 (dd, J = 15.0, 4.7 Hz, 1H),2.18 (dd, J = 15.0, 8.3 Hz, 1H), 2.10 (m, 2H),2.00 (dt, J = 7.2, 7.2 Hz, 2H), 1.35 (tq, J = 7.7, 7.2 Hz, 2H), 0.86 (t, J = 7.7 Hz, 3H) ppm. ^{13}C NMR (226 MHz, DMSO-d_6) δ 172.94, 132.25, 130.74, 130.35, 130.08, 130.04, 127.43, 67.23, 41.86, 40.17, 35.00, 34.05, 22.02, 13.55 ppm. ESI-HRMS (*m/z*): $[M-H]^-$ calcd. for $C_{14}H_{22}O_3$ 237.1496; found 237.1496. $[\alpha]_D^{20}$ -9.1° (*c* 1, $CHCl_3$).

Compound 3 Data:

^{1}H NMR (900 MHz, CD_3OD) δ 6.01 (m, 2H), 5.98 (d, J = 1.6 Hz, 1H), 5.57 (m, 1H), 5.54 (m, 1H), 5.53 (dt, J = 15.4, 6.6 Hz, 1H), 5.49 (dt, J = 15.4, 6.6 Hz, 1H), 4.95 (d, J = 4.8 Hz, 1H), 4.30 (dd, J = 8.6, 7.7 Hz, 1H), 4.04 (m, 1H), 3.97 (dd, J = 8.6, 4.8 Hz, 1H), 3.89 (dd, J = 3.7, 1.6 Hz, 1H),

3.82 (dd, J = 7.7, 1.7 Hz, 1H), 3.70 (dd, J = 9.6, 3.6 Hz, 1H), 3.67 (dq, J = 9.8, 6.2 Hz, 1H), 3.64 (ddd, J = 7.1, 5.9, 1.7 Hz, 1H), 3.60 (dd, J = 10.8, 5.9 Hz, 1H), 3.59 (dd, J = 10.8, 7.1 Hz, 1H), 3.41 (dd, J = 9.8, 9.6 Hz, 1H), 2.78 (t, J = 6.2 Hz, 2H), 2.57 (dd, J = 15.3, 4.0 Hz, 1H), 2.40 (dd, J = 15.3, 8.8 Hz, 1H), 2.23 (m, 2H), 2.03 (dt, J = 7.3, 7.0 Hz, 2H), 1.41 (tq, J = 7.4, 7.3 Hz, 2H), 1.26 (d, J = 6.2 Hz, 3H), 0.91 (t, J = 7.4 Hz, 3H) ppm; ^{13}C NMR (226 MHz, CD_3OD) δ 171.51, 133.48, 132.88, 132.47, 131.82, 130.74, 127.59, 103.51, 93.80, 82.61, 79.17, 78.46, 74.52, 73.58, 72.52, 71.51, 71.33, 69.29, 64.34, 42.57, 41.45, 36.58, 35.77, 23.66, 17.94, 14.00 ppm; ESI-HRMS (*m/z*): $[M-H]^-$ calcd. for $C_{26}H_{42}O_{12}$ 545.2604; found 545.2600.

3-HTA Data:

^{1}H NMR (900 MHz, $CDCl_3$) δ 4.01 (m, 1H), 2.56 (dd, J = 16.6, 3.0 Hz, 1H), 2.46 (dd, J = 16.6, 9.1 Hz, 1H), 1.53 (m, 1H), 1.44 (m, 2H), 1.35 – 1.22 (m, 17H), 0.86 (t, J = 7.2 Hz, 3H) ppm. ^{13}C NMR (226 MHz, $CDCl_3$) δ 176.28, 68.22, 40.95, 36.76, 32.13, 29.86, 29.84, 29.79, 29.77, 29.69, 29.56, 25.65, 22.91, 14.35 ppm. ESI-HRMS (*m/z*): $[M-H]^-$ calcd. for $C_{14}H_{28}O_3$ 243.1966; found 243.1933. $[\alpha]_D^{20}$ -15.7° (*c* 1, $CHCl_3$).

Supplementary References

1. Yadav, G., Gokhale, R. S. & Mohanty, D. Computational Approach for Prediction of Domain Organization and Substrate Specificity of Modular Polyketide Synthases. *Journal of Molecular Biology* **328,** 335–363 (2003).

2. Buntin, K., Irschik, H., Weissman, K. J., Luxenburger, E., Blöcker, H. & Müller, R. Biosynthesis of Thuggacins in Myxobacteria: Comparative Cluster Analysis Reveals Basis for Natural Product Structural Diversity. *Chemistry & Biology* **17,** 342–356 (2010).
