# Supplementary material for: The industrial anaerobe Clostridium acetobutylicum uses polyketides to regulate cellular differentiation
Source: Nat Commun. 2017 Nov 15;8:1514. doi: 10.1038/s41467-017-01809-5 (PMC5686105; doi:10.1038/s41467-017-01809-5)
Supplement: Supplementary file 2 — Descriptions of Additional Supplementary Files [file 41467_2017_1809_MOESM2_ESM.pdf]

Descriptions of Additional Supplementary File

File Name: Supplementary Dataset 1
Descriptions: Results of RNA-Seq analysis comparing wild-type C. acetobutylicum and Δpks. Gene annotation based on NCBI designation. STRING group refers to the cellular processes designated in Figure 3. Fold change and p-value refer to differences in gene expression between wild-type and Δpks strains (unpaired t-test, $n = 3$). Fold change < 0 signifies the gene was downregulated in Δpks relative to wild-type, while upregulated genes have fold change > 0. The last column provides the false discovery rate (FDR)-corrected p-values, calculated as described in the main text.
